# Active With Whom? Examining the Social Context of Physical Activity in Individuals After Stroke and Their Partners

**DOI:** 10.3389/fpubh.2021.754046

**Published:** 2021-09-29

**Authors:** Theresa Pauly, Maureen C. Ashe, Rachel Murphy, Denis Gerstorf, Wolfgang Linden, Kenneth M. Madden, Christiane A. Hoppmann

**Affiliations:** ^1^Department of Psychology, University of Zurich, Zurich, Switzerland; ^2^University Research Priority Program “Dynamics of Healthy Aging,” University of Zurich, Zurich, Switzerland; ^3^Center for Hip Health and Mobility, University of British Columbia, Vancouver, BC, Canada; ^4^Department of Family Practice, University of British Columbia, Vancouver, BC, Canada; ^5^BC Cancer Research Centre, Vancouver, BC, Canada; ^6^School of Population and Public Health, University of British Columbia, Vancouver, BC, Canada; ^7^Department of Psychology, Humboldt University Berlin, Berlin, Germany; ^8^Department of Psychology, University of British Columbia, Vancouver, BC, Canada; ^9^Department of Medicine, University of British Columbia, Vancouver, BC, Canada

**Keywords:** physical activity, close relationships, older adults, stroke, accelerometer, daily life research, MVPA, couples

## Abstract

Engaging in regular moderate-to-vigorous intensity physical activity (MVPA) is crucial to reduce future health risk for individuals living with the effects of a stroke and their partners. Although numerous studies point to the importance of social factors in physical activity engagement, little is known about with whom individuals after stroke and their partners engage in physical activity with and whether different physical activity companions are uniquely associated with MVPA. Eighty-nine community-dwelling individuals after stroke (*M*_age_ = 68.64, *SD* = 10.44; 74% male) and 83 partners (*M*_age_ = 66.04, *SD* = 9.91; 24% male) completed 14 consecutive days of daily life assessments that included wearing physical activity monitors (accelerometers) and self-reporting physical activity companions (*n* = 1,961 days). Results show that average levels of MVPA were correlated between partners (*r* = 0.38), as were day-to-day MVPA fluctuations (*r* = 0.34). Importantly, for individuals after stroke, being active with their partner, but not with any other physical activity companion, was linked with elevated daily MVPA. In contrast, for partners of individuals after stroke, engaging in physical activity with a variety of different companions (partner, other family member, friend, colleague) was each associated with higher MVPA in daily life. For both individuals after stroke and their partners being active by oneself (without a companion) on a given day was not associated with elevated MVPA. Findings suggest that interventions that promote physical activity engagement should consider the role of meaningful others, with the partner being particularly key for individuals living with chronic health conditions.

## Introduction

Stroke is a major cause of morbidity and mortality (1, 2). It profoundly alters the life of the survivor and their close others, and results in costly health care utilization (3–5). Health behaviors, in particular physical activity, reduce initial stroke risk and help prevent (or reduce) future risk of stroke (6). In fact, an estimated 36% of stroke incidence is attributable to insufficient physical activity levels [population attributable risk; (7)]. Current guidelines recommend at least 150-300 min of moderate or 75-150 min of vigorous physical activity per week to achieve substantial health benefits (8). However, given that only about 20% of adults worldwide meet physical activity guidelines (8, 9), it is pivotal to address psycho-social predictors of this key health behavior. Most research on psychological determinants of health behaviors is focused at the individual-level. Yet, physical activity is, to a great extent, shaped by its social context. In particular, life partners influence each other's health behaviors (10, 11), and other companions may also play a role in shaping physical activity levels. Therefore, our objective was to examine with whom individuals after stroke and their partners engage in physical activity and whether day-to-day variation in moderate-to-vigorous physical activity (MVPA) differs by type of physical activity companion.

### Physical Activity in Individuals After Stroke and Their Partners

Physical activity is commonly categorized into at least three categories, based on energy expenditure thresholds: Sedentary behavior as ≤1.5 metabolic equivalents (MET); light physical activity as 1.5 to 3 MET; and MVPA as ≥3 MET (12, 13). Although there are clear benefits to physical activity for cardiovascular health (14), people with stroke tend to exhibit little time in MVPA and high sedentary time (15, 16). A recent review (16) concluded that individuals after stroke were less active than individuals with other chronic conditions, such as diabetes and chronic obstructive pulmonary disease (COPD); and that they took half the amount of steps (4,078 vs. 8,338 steps/day) and expended half the daily energy on physical activity (1257.3 vs. 2108.9 kcal/day), as compared with healthy individuals. This is concerning because physical activity, especially MVPA, plays a crucial role for stroke recovery to mitigate residual effects of stroke and to manage future cardiovascular risk (14, 17).

Recent health psychological research highlights the important role of the social environment for physical activity engagement (18–20). Average physical activity levels are linked in life partners, as are changes in physical activity over time (11, 21–23). Partners of individuals after stroke often take on caregiving responsibilities (24–26). Caregivers are at increased risk for developing chronic diseases themselves, due to high psychological stress, physical exertion, and a lower likelihood of engaging in health-promoting behaviors (27–29). Thus, engaging in an adequate amount of physical activity to mitigate health risk is not only important for individuals after stroke, but also for their partners. To better understand how social resources can be leveraged to promote physical activity in couples post stroke, it is key to systematically investigate the everyday social context of physical activity in individuals after stroke and their partners.

### Physical Activity Companions

Although some individuals prefer to exercise by themselves, a significant number of adults prefer to engage in physical activity with a companion (30, 31). In fact, for individuals after stroke it might be important not to exercise alone for safety reasons [e.g., due to risk of falling; (32)]. Engaging in physical activity with a companion, such as a life partner, has been associated with physical activity initiation, higher physical activity levels, and better adherence to exercise regimens in community-dwelling samples (33–38). For example, in a representative sample of 2025 young, middle-aged, and older adults, those who reported that they can easily find an exercise partner had a 47% higher likelihood of meeting physical activity guidelines than those without exercise partners (36). In addition, Giles-Corti and Donovan (39) showed that social factors (e.g., club membership) were more closely associated with physical activity levels than physical features of the environment (e.g., spatial access to recreational facilities) in young to middle-aged adults. Thus, based on retrospective self-reports, there is initial evidence that individuals often like to engage in physical activity with a companion. Yet, very little is known about the social context of physical activity in an everyday setting. As the next step, it would be important to move from retrospective self-reports to objective physical activity monitoring, so as to comprehensively examine the nature and predictors of how adults with chronic illness and their partners are physically active.

### The Current Study

The current study aims to fill this gap, by collecting self-report data on physical activity companions and objective data on physical activity of at least moderate intensity (MVPA) for a 14-day period as individuals after stroke and their partners go about their daily lives. Based on previous studies showing links in couples' physical activity levels (40, 41), we expected that MVPA would be interrelated between individuals after stroke and their partners, both in terms of their average MVPA levels as well as in their day-to-day fluctuations in MVPA. Furthermore, we aimed to investigate whether different types of physical activity companions would be uniquely associated with daily MVPA in individuals after stroke, as compared to their partners. In our analysis, we control for variables known to be associated with physical activity, including age, sex, education, and gait speed (42, 43).

## Materials and Methods

### Participants and Procedure

The sample included 89 community-dwelling individuals after stroke aged 33-88 years (*M*_age_ = 68.64, *SD* = 10.44; 74% male; 36% with college degree; 83% White) and 83 spouses or common-law partners of individuals after stroke (aged 37-83 years; *M*_age_ = 66.04, *SD* = 9.91; 24% male; 34% with college degree; 81% White) from rural and metropolitan areas in southern British Columbia, Canada. Participants were part of a larger study on health behaviors in couples post stroke. Study inclusion criteria were: the ability to communicate verbally, read newspaper-sized print, and being able to independently walk ≥10 m (with/without walking aid).

Out of 244 individuals who expressed interest in the study by phone or email, 139 met eligibility criteria. Out of the 139 eligible couples, 101 consented to participate (*n* = 202 participants). One couple was a pilot couple to test feasibility of the study protocol, 22 individuals dropped out of the study, data of one person was excluded because of data fidelity concerns, and one couple did not have complete daily diary data due to technical issues. Furthermore, three participants did not provide sufficient physical activity data and were removed during data cleaning (see below). For the purpose of this study, we retained individuals in the sample even if the respective partner provided incomplete data. This resulted in a final sample of 172 participants, of whom both partners' data were complete for 83 couples. Almost one quarter of the sample (24%) lived more than a 2 h drive away from the next cardiac care center. On a scale of 1 “poor” to 5 “excellent,” individuals after stroke rated their health as 2.57 (*SD* = 0.90), on average, whereas partners' average health rating was 3.20 (*SD* = 0.85). Most partners were in a long-term relationship (*M* relationship duration: 35.00 years, *SD* = 16.08, range: 2-66 years) and retired (individuals after stroke: 75%, partners: 64%), and they reported high relationship satisfaction (*M* = 4.40 out of 5, *SD* = 0.61).

As part of a 14-day time-sampling phase, participants completed brief daily electronic surveys on tablets (every morning and evening) and wore a physical activity monitor during waking hours. Participants showed good adherence to the daily protocol (*M* = 12.5/14 days of completed evening questionnaires, *SD* = 2.7) and reported that the study period was typical for their daily lives (*M* = 4.03 out of 5, *SD* = 1.14). For taking part in the study, participants were offered the choice to keep one tablet per couple or CAD $100 each. The study was approved by the University of British Columbia ethics board and participants provided informed consent.

### Measures

#### MVPA

MVPA was measured using a hip-worn accelerometer (GT3X+, ActiGraph, Pensacola, US). Raw physical activity data were processed using ActiLife Version 6.6.2. Non-wear time was defined as at least 90 consecutive min of no detected activity (44). Days with <10 h of valid wear time and participants with less than 3 valid wear days were excluded [*n* = 3; (45)]. To classify intensity of movement (moderate to vigorous), we used a cut-point of ≥1,952 counts/min, as suggested by Freedson et al. (46).

#### Physical Activity Companions

Every evening, participants self-reported with whom they had engaged in MVPA that day: (a) alone, (b) partner, (c) other family member/s, (d) friend, (e) colleague/co-worker, and/or (f) other. Participants were able to select one or multiple options. MVPA was explained to participants as the following: “Moderate to high intensity physical activity is any type of activity that makes your heart beat more. For example, that could be brisk walking, swimming, or biking.”

#### Covariates

Participants also self-reported their age, sex (female or male), and education (no college degree, or college degree). To assess gait speed (47), participants were instructed to walk a distance of 6 m in their usual walking speed twice. Gait speed was computed by the average time across the two walking trials from the 1 m to the 5 m mark and converted to meters walked per second (m/s). Information on age was missing for one individual and replaced with the respective partner's age.

### Statistical Analyses

Data were analyzed using multilevel models [*R* lme4 package; (48)] with days (level 1) nested within participants (level 2). Models adjusted for age, sex, day in study, education, gait speed, and person means of daily measures. Continuous variables were centered on the sample mean. Dichotomous variables were left uncentered (sex, education, physical activity companions). Thus, the intercept reflects the average objectively measured MVPA in minutes of a typical male participant in the sample without a college degree on a day with no self-reported physical activity of at least moderate intensity (alone or with a companion). Explained variance in multi-level models was calculated using the MuMIn package (49). Repeated measures correlation was calculated using the rmcorr package (50).

## Results

Descriptive statistics and bivariate correlations of sample characteristics can be found in Table 1. Older age (individuals after stroke: *r* = –.28, *p* = 0.008; partners: *r* = −0.30, *p* = 0.005) and lower gait speed (individuals after stroke: *r* = 0.53, *p* < 0.001; partners: *r* = 0.24, *p* = 0.026) were associated with less MVPA. In individuals after stroke, older age was also associated with lower gait speed (*r* = −0.34, *p* = 0.001). Only 25% of individuals after stroke engaged in at least 150 min of MVPA per week. Comparatively, 45% of partners of individuals after stroke engaged in at least 150 min of MVPA per week.

**Table 1:** Descriptive statistics and correlations of sample characteristics (*N* = 172 Participants).

		**Individuals after stroke (*****n*** **=** **89)**		**Partners (*****n*** **=** **83)**					
	**Variables**	**M or %**	**(SD)**	**M or %**	**(SD)**	**1**	**2**	**3**	**4**	**5**
1	Age	68.64^a^	10.44	66.04^a^	9.91	0.86^**^	0.16	0.07	−0.34^**^	−0.28^**^
2	Sex (male)	74%^a^		24%^a^		0.08	–1.00^**^	0.23^*^	0.01	0.14
3	Education (college degree)	36%		34%		−0.12	0.07	0.31^**^	0.10	0.16
4	Gait Speed (m/s)	0.94^a^	0.34	1.21^a^	0.24	−0.06	−0.03	0.10	**0.19**	0.53^**^
5	MVPA (min/day)	14.76^a^	19.81	21.94^a^	17.96	−0.30^**^	0.05	0.21	0.24^*^	0.38^**^

*MVPA, moderate-to-vigorous physical activity. Gait speed is meters walked per second. Correlations for individuals after stroke are presented above the main diagonal, correlations for partners are presented below the main diagonal, and intercorrelations between individuals after stroke and their partners are displayed in bold in the main diagonal*.

* *p < 0.05*,

** *p < 0.01*.

a *Mean differences between individuals after stroke and their partners are significant*.

### Physical Activity in Individuals After Stroke and Their Partners

Individuals showed considerable fluctuations in MVPA in daily life, with 46% (individuals after stroke) to 51% (partners) of variance in MVPA being attributed to differences between days within individuals (see Supplementary Figure 1). Individuals after stroke engaged in an average of 14.76 min of MVPA/day (range: 0.20-99.14, *SD* = 19.81)^1^, which was significantly less than their partners (*M* = 21.94 min/day, range: 0.60-63.40, *SD* = 17.96; *t*(81) = −3.01, *p* = 0.003). MVPA was correlated among individuals after stroke and their partners on a between- and a within-person level. Specifically, individuals after stroke who engaged in more average MVPA also tended to have a partner who engaged in more average MVPA (*r*_between_ = 0.38, *p* < 0.001). Additionally, if one participant engaged in a higher amount of MVPA than was typical for them on a given day, their partner engaged in more MVPA, too (*r*_repeated_ = 0.34, *p* < 0.001).

### Physical Activity Companions

**Figure 1:**
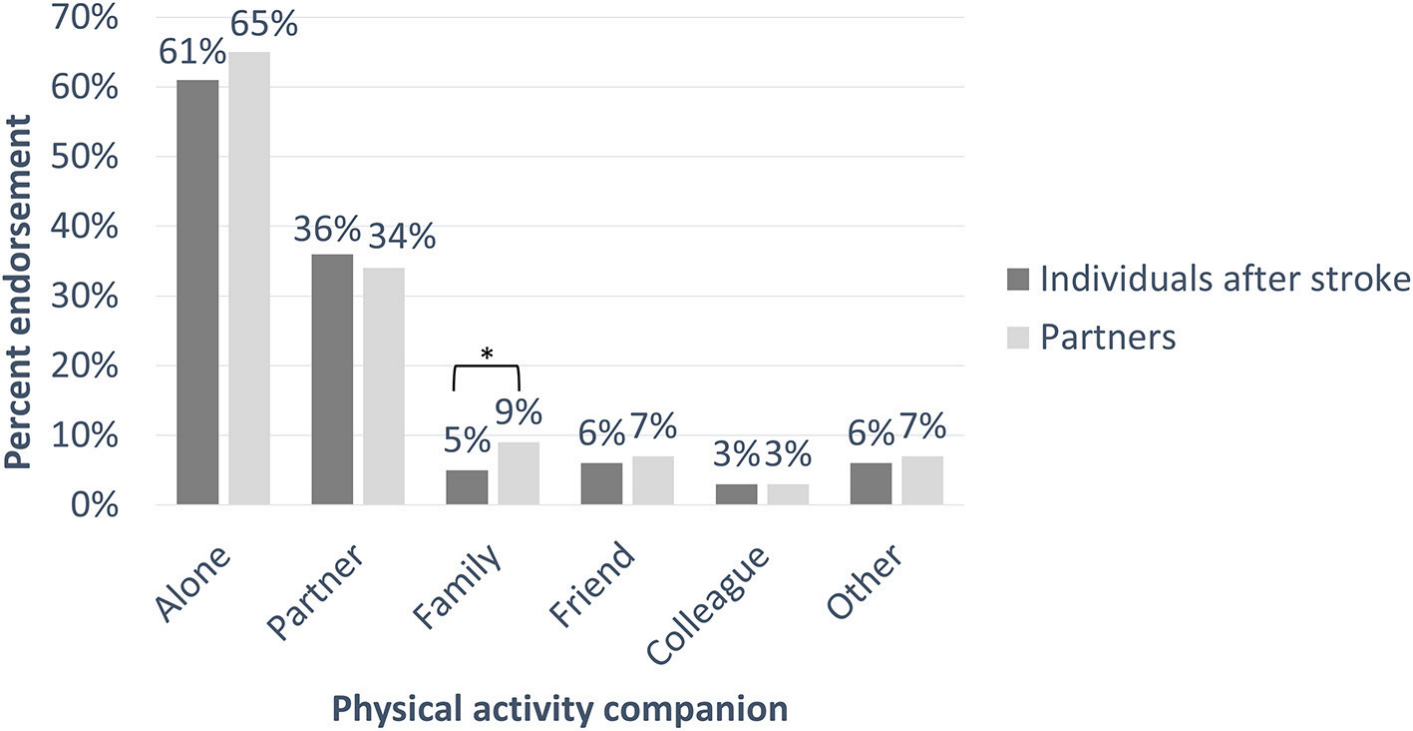
The percentage of days on which participants reported engaging in moderate-to-vigorous physical activity (MVPA, as measured by accelerometers) by themselves or with different companions for individuals after stroke and their partners. Participants were able to select one or multiple options. *p < 0.05.

Figure 1 and Supplementary Table 1 display the percentage of days individuals reported having engaged in MVPA by themselves or with different companions (multiple answers possible). Most participants reported engaging in physical activity alone (individuals after stroke: 61% of days, partners: 65%) or with their partner (individuals after stroke: 36% of days, partners: 34%). Less frequently, participants engaged in physical activity with a family member (individuals after stroke: 5% of days, partners: 9%), friend (individuals after stroke: 6% of days, partners: 7%), colleague (individuals after stroke: 3% of days, partners: 3%), or other person (individuals after stroke: 6% of days, partners: 7%).

**Figure 2:**
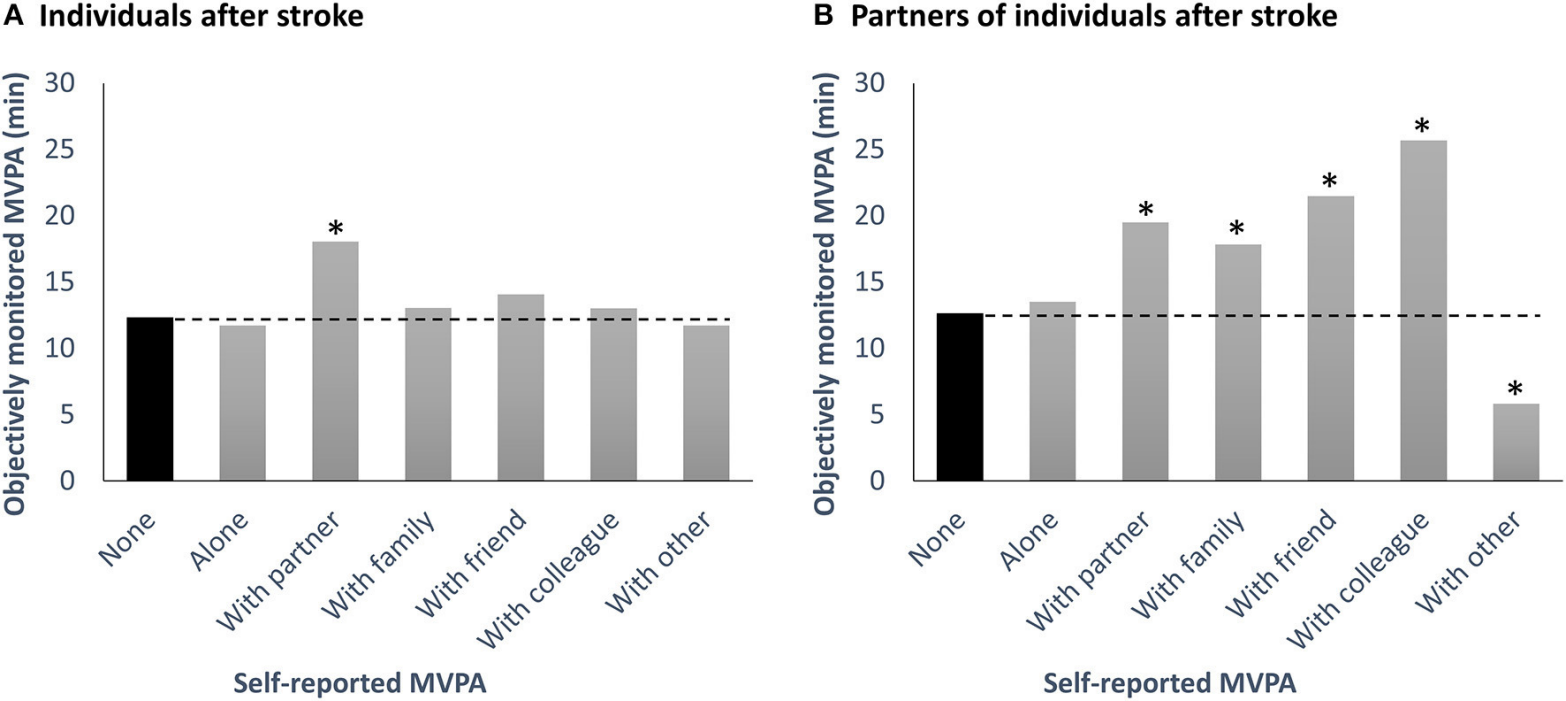
Model-implied moderate-to-vigorous physical activity (MVPA) levels as measured by accelerometer on days without any self-reported physical activity and on days when physically active by oneself or with different companions for individuals after stroke **(A)** and their partners **(B)**. Asterisks (*) denote a significant difference (*p* < 0.05) of MVPA levels on days when active with the respective companion or by oneself, as compared with days without any self-reported MVPA. For individuals after stroke, engaging in physical activity with the partner was associated with 6 min more MVPA/day (which would translate to 30 min more MVPA/week if active together for 5 days). For partners of individuals after stroke, engaging in physical activity with the partner was associated with 7 min more MVPA/day (which would translate to 35 min more MVPA/week if active together for 5 days). MVPA, Moderate-to-vigorous physical activity.

We next examined time-varying associations between self-reported MVPA by oneself or with different companions and objectively measured MVPA in multi-level models (see Table 2; Figure 2). On days on which participants reported to be physically active by themselves, neither individuals after stroke nor their partners showed significantly higher MVPA than usual. For individuals after stroke, engaging in physical activity with the partner was linked with significantly increased MVPA (*b* = 5.72, *SE* = 1.67, *p* < 0.001). Specifically, individuals after stroke engaged in 6 min more MVPA on days when they were active with their partner. No other type of companion was significantly related to MVPA in individuals after stroke. For partners of individuals after stroke, engaging in physical activity with their partner (*b* = 6.85, *SE* = 1.56, *p* < 0.001), another family member (*b* = 5.18, *SE* = 2.42, *p* < 0.032), a friend (*b* = 8.84, *SE* = 2.54, *p* < 0.001), and a colleague (*b* = 13.04, *SE* = 4.89, *p* = 0.008) were all associated with greater than usual daily MVPA. However, when partners were active with an unspecified other person there was lower than usual MVPA (*b* = −6.82, *SE* = 2.82, *p* = 0.016).

**Table 2:** Results from Multilevel Models Examining MVPA Using Restricted Maximum Likelihood Estimation (*N* = 172 Participants, *n* = 1,961 Days).

	**Individuals after stroke (*****n*** **=** **89)**				**Partners of individuals after stroke (*****n*****=** **83)**		
**Variable**	**B (SE)**	**95% CI**	***p***		**B (SE)**	**95% CI**	***p***
**Fixed Effects**							
Intercept	**12.35 (4.25)**	**[4.40;20.28]**	**0.004**		**12.65 (3.08)**	**[6.89;18.41]**	** <0.001**
Day of Study	−0.15 (0.13)	[−0.40;0.11]	0.270		−0.18 (0.13)	[−0.44;0.08]	0.180
Age	−0.34 (0.20)	[−0.71;0.02]	0.081		−**0.62 (0.21)**	**[**–**1.00;** –**0.23]**	**0.004**
Sex	5.94 (4.55)	[−2.47;14.39]	0.195		3.19 (4.38)	[−4.89;11.27]	0.469
Education	3.38 (4.08)	[−4.18;10.95]	0.410		5.22 (3.96)	[−2.08;12.54]	0.192
Gait Speed	**29.23 (5.98)**	**[18.17;40.35]**	** <0.001**		**18.24 (8.18)**	**[3.17;33.35]**	**0.029**
PhyComp Alone	−0.61 (1.70)	[–3.93;2.72]	0.722		0.88 (1.66)	[−2.38;4.13]	0.599
PhyComp Partner	**5.72 (1.67)**	**[2.47;8.98]**	** <0.001**		**6.85 (1.56)**	**[3.80;9.90]**	** <0.001**
PhyComp Family	0.70 (2.92)	[–5.00;6.40]	0.811		**5.18 (2.42)**	**[0.46;9.90]**	**0.032**
PhyComp Friend	1.71 (2.57)	[−3.32;6.73]	0.507		**8.84 (2.54)**	**[3.87;13.80]**	** <0.001**
PhyComp Colleague	0.67 (3.62)	[−6.41;7.74]	0.854		**13.04 (4.89)**	**[3.49;22.59]**	**0.008**
PhyComp Other	−0.63 (3.08)	[−6.65;5.40]	0.839		−**6.82 (2.82)**	**[**–**12.34;** –**1.31]**	**0.016**
**Random Effects**							
Intercept	**15.84**	**[12.54;17.47]**	** <0.001**		**15.49**	[12.09;17.09]	** <0.001**
Level-1 Residual	**16.07**	**[15.29;16.78]**	** <0.001**		**16.92**	[16.11;17.66]	** <0.001**
**Model Fit**							
Deviance	8282.3				8494.5		

*B, unstandardized regression coefficient. SE, standard error. CI, confidence interval. PhyComp, Physical activity companion. Sex was coded 0, female; 1, male. Eduction was coded 0, no college degree; 1, college degree. Gait speed is meters walked per second. Bold font denotes significant regression coefficients. All continuous variables were grand mean-centered. Models also control for person average endorsements of physical activity companions (not shown for simplicity)*.

With respect to co-variates, MVPA did not significantly differ by sex or education level in individuals after stroke or their partners. Furthermore, objectively measured MVPA did not differ by day of study. However, participants with lower gait speed showed lower MVPA levels (individuals after stroke: *b* = 29.23, *SE* = 5.98, *p* < 0.001; partners: *b* = 18.24, *SE* = 8.18, *p* = 0.029). Additionally, older age was linked with less daily MVPA in partners (*b* = −0.62, *SE* = 0.21, *p* = 0.004). Explained variance in MVPA by fixed effects only was 23% for individuals after stroke and 16% for their partners; explained variance in MVPA by fixed and random effects combined was 61% for individuals after stroke and 55% for their partners.

## Discussion

This study examined levels of objectively measured MVPA in the everyday lives of individuals after stroke and their partners. Our type of assessment protocol improves the validity of measurement because it does not rely on global, retrospective memory (13, 52). We found that average levels of MVPA co-varied in couples. Furthermore, as predicted, partners had more MVPA on days when their partners also had higher MVPA. Importantly, we noted that *for partners* engaging in physical activity with a variety of different companions was associated with higher MVPA in daily life (e.g., family member, friend). However, for *individuals after stroke*, engaging in physical activity with someone else was only associated with increased MVPA if the companion was the partner.

### Physical Activity in Individuals After Stroke and Their Partners

As hypothesized, partners of individuals after stroke with lower average MVPA levels had lower average MVPA levels themselves (moderate to large effect size). In line with prior research (16), a minority (25%) of individuals after stroke engaged in at least 150 min of MVPA per week. Partners of individuals after stroke were relatively active as compared to population averages (9, 53), however still less than half (45%) accumulated > 150 min of MVPA per week. We also investigated how the amount of MVPA was interdependent between partners in a day-to-day context. As expected and in line with prior research demonstrating co-varying physical activity in community-dwelling and clinical samples on a daily and hour-by-hour basis (40, 41, 54), we found that MVPA was higher than usual in individuals after stroke on days when their partner's MVPA was also higher (moderate effect size). A close linkage in health behaviors in couples might carry both health-enhancing as well as health-compromising ramifications for both partners' functioning^2^. For example, it might mean that any declines in physical activity resulting from stroke-related mobility impairments could carry over to the partner (55). Considering the health ramifications associated with insufficient physical activity levels (56), it is thus of major public health importance to identify social change agents that can help individuals after stroke and their partners to be more active. In this study, we go beyond looking at life partners to also consider other physical activity companions.

### Physical Activity Companions

Participants engaged in physical activity alone most of the days that they were active (61-66%). We found for both individuals after stroke as well as their partners that being active by oneself on a given day, without any companion, was not significantly associated with higher MVPA. In contrast to being active alone, being active with the partner (34-36% of days) was linked with higher daily MVPA in both individuals after stroke and their significant others. This extends previous research conducted in younger to middle-aged samples showing that exercising with a companion is linked with higher exercise intensity (35) and duration (57). Such findings support recent health behavior innovations recognizing the importance of the partnership for physical activity (58, 59). Indeed, interventions including the partner may be more effective in enhancing physical activity than individual-based interventions (60).

Although self-reported physical activity companions (e.g., friend) did not largely differ between partners, their link with engaging in MVPA on a given day did. For individuals after stroke, daily MVPA was higher only on days when they reported being active with their partner; it was not higher when they reported being active with any other physical activity companion. In contrast, partners of individuals after stroke also showed greater MVPA on days when they were active with another family member, a friend, and a colleague. There are several reasons why the partnership might play a uniquely important role for MVPA post stroke. First, when faced with limited cognitive resources and physical functioning, individuals after stroke might invest more in maintaining their closest and most intimate relationship (61, 62). Second, the partner might be most familiar with one's capabilities and thus better able to provide the needed type of support and encouragement to achieve an appropriate intensity level (63). Third, trust might be essential and particularly high in romantic relationships, thereby helping to reduce barriers to physical activity like fear of falling (64). For partners of individuals after stroke, being active with a person who was not captured in the categories of partner, family, friend, or colleague was linked with lower daily MVPA. This “other”-category was infrequently identified by participants and likely captured a range of individuals with an unknown social relationship to participants. We therefore caution against overinterpreting this finding.

A recent study suggests that the link between being active with a companion and increased physical activity might be explained by individuals experiencing more positive affect when being active with others than alone (57). Engaging in physical activity with a partner might not just be beneficial in that it increases physical activity duration or intensity, but it might also improve relationship quality (65). For example, joint physical activity has been associated with relationship satisfaction, positive marital events, and closeness (41, 65, 66).

### Implications of Findings for Public Health

A number of conceptual and theoretical models recognize the inherently social nature of health, health behaviors, and disease management (67–69). Even though there is ever-increasing empirical support for these theories, pointing to the crucial importance of the social environment for physical activity engagement (11, 18, 22), such notions have yet to be implemented in health care and rehabilitation. For example, programs to enhance physical activity might be more effective when targeting both partners in a couple rather than just one (34, 60). Our findings dovetail with this idea, suggesting that recruiting the partner as a change agent might be particularly important in individuals after stroke. This could include involving partners of individuals after stroke in rehabilitation, for example by joint goal setting and planning joint exercise (37, 70). Overall, a more holistic approach that pays tribute to the close linkage of health-promoting and health-compromising behaviors in life partners could help optimize health and well-being, particularly in adults living with a chronic disease.

### Strengths, Limitations, and Future Directions

As a strength, the current study collected self-report data on physical activity companions and objectively measured movement intensity in the daily lives of individuals after stroke and their partners over 14 days. Walking capacity (gait speed) and average MVPA per day in individuals after stroke who participated in our study were comparable to population averages (51, 71). Furthermore, the sample was relatively diverse with respect to education and living environments (rural vs. metropolitan). As a limitation, the hip-worn accelerometers might not have been able to accurately capture the intensity of some activities, including cycling (72) or swimming (participants were instructed to take off the device during water-based activities). Furthermore, individuals after stroke mostly comprised male participants in our sample, whereas partners were mostly female. We adjusted for sex in our models, but larger samples are needed to examine how differences in social network structures and social support provision between men and women (73–75) might shape the influence of physical activity companions. In addition, most participants were older adults, retired, and reported relatively high relationship satisfaction. Future studies should investigate the role of companions for physical activity intensity in younger adults, in individuals after stroke who are still working, and in couples who report lower relationship quality. The prevalences of physical activity companions other than the partner were relatively low (3-8%). Thus, these findings have to be interpreted with caution. Future research could examine whether results are specific to individuals after stroke or generalize to adults living with other chronic diseases, including osteoarthritis and diabetes (76, 77). Finally, future studies could also collect data on *type* of physical activity on a daily level. It might be that individuals more likely engage in low intensity types of activities when alone (e.g., walking), whereas they more likely engage in high intensity types of activity (e.g., riding a bicycle) when with others.

## Conclusions

The experience of a stroke often presents a major disruption to couples' daily lives (5). Because physical activity plays a crucial role in mitigating future cardiovascular risk in individuals after stroke and their partners (14), identifying social change agents that can help promote physical activity is key, particularly physical activity of moderate-to-high intensity. In line with prior research, we found partners' physical activity levels to be closely intertwined, at a between-person level (average MVPA) and a within-person level (daily MVPA fluctuations). We further investigated the role of different self-reported physical activity companions for objectively measured MVPA. Results showed that for partners of individuals after stroke engaging in physical activity with the partner, another family member, a friend, or a colleague was associated with higher MVPA in daily life. For individuals after stroke, engaging in physical activity with someone else was only associated with increased MVPA if the companion was the partner. Neither individuals after stroke nor their partners engaged in more daily MVPA on days when they self-reported only being physically active by themselves, without any companion. Findings underline the pivotal role of partners for physical activity in adults living with chronic illness. Specifically, they suggest that public health efforts that pay tribute to the fundamental social nature of MVPA engagement could constitute a promising future direction to enhance health in couples post stroke.

## Data Availability Statement

The raw data supporting the conclusions of this article will be made available by the authors, without undue reservation.

## Ethics Statement

The studies involving human participants were reviewed and approved by the Clinical Research Ethics Board, The University of British Columbia. The patients/participants provided their written informed consent to participate in this study.

## Author Contributions

CH, MA, RM, DG, WL, and KM designed and directed the project. CH supervised the work. TP recruited participants, coordinated data collection, performed the data analysis, and wrote the first draft of the manuscript. All authors commented on the manuscript and contributed to the final version of the manuscript.

## Funding

This work was supported by a grant from the Heart and Stroke Foundation of Canada (G-16-00012717 to CH, MA, RM, DG, WL, and KM).

## Conflict of Interest

The authors declare that the research was conducted in the absence of any commercial or financial relationships that could be construed as a potential conflict of interest.

## Publisher's Note

All claims expressed in this article are solely those of the authors and do not necessarily represent those of their affiliated organizations, or those of the publisher, the editors and the reviewers. Any product that may be evaluated in this article, or claim that may be made by its manufacturer, is not guaranteed or endorsed by the publisher.

## Footnotes

^1^This number compares to average MVPA per day as measured by accelerometer for Canadian adults living with a chronic disease [15.6 min/day; (51)].

^2^Pauly T, Gerstorf D, Wahl H-W, Hoppmann CA. A developmental-contextual model of dyadic synchrony across adulthood and old age. *Psychol Aging*. (under review).
