# Supplementary material for: Active With Whom? Examining the Social Context of Physical Activity in Individuals After Stroke and Their Partners
Source: Front Public Health. 2021 Sep 29;9:754046. doi: 10.3389/fpubh.2021.754046 (PMC8511423; doi:10.3389/fpubh.2021.754046)
Supplement: Supplementary file 1 [file Data_Sheet_1.DOCX]

**S-Table 1**

*Physical Activity Companions in Individuals after Stroke and Their Partners (N = 172 Participants)*

|  | Individuals After Stroke (n = 89) | Partners of Individuals After Stroke (n = 83) | Difference Between Partners |
| --- | --- | --- | --- |
| Physical Activity Companion | *%* / n_days_ | % / n_days_ | *p* |
| Alone | 61% / 596 days | 65% / 640 days | .226 |
| Partner | 36% / 380 days | 34% / 350 days | .483 |
| Family | 5% / 46 days | 9% / 83 days | .012 |
| Friend | 6% / 60 days | 7% / 72 days | .533 |
| Colleague | 3% / 28 days | 3% / 30 days | .975 |
| Other | 6% / 47 days | 7% / 72 days | .565 |

*Note.* n = number.

**S-Figure 1**

*Within-person fluctuations of MVPA across the study period*

**
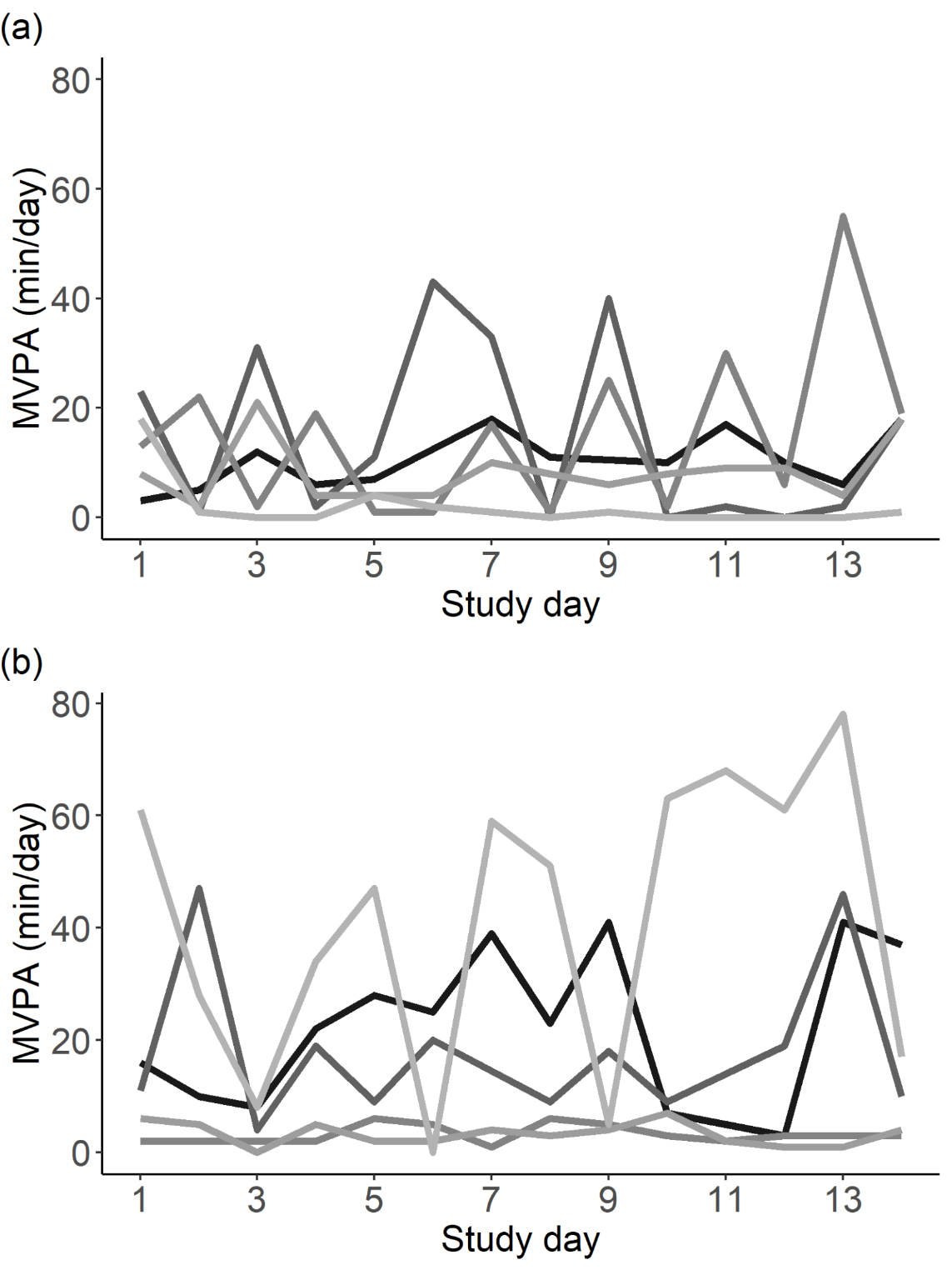
**

*Note*. The figure displays MVPA levels of five randomly selected individuals after stroke (a) and of partners of individuals after stroke (b) across the 14 study days. Each line represents one participant. MVPA = moderate-to-vigorous physical activity. It can be obtained that MVPA showed significant day-to-day fluctuations.
